# Behavioral Innovations to Access Abortion Post-Dobbs: A Qualitative Thematic Analysis of Reddit’s r/abortion Community in 2022

**DOI:** 10.1177/00469580251351192

**Published:** 2025-06-27

**Authors:** Eliza Dolgins, Lindsay Parham, Karen Weidert, Emma Anderson, Coye Cheshire, Ndola Prata, Elizabeth Pleasants

**Affiliations:** 1 University of California, Berkeley, Berkeley, CA, USA; 2 University of North Carolina, Chapel Hill, Chapel Hill, NC, USA

**Keywords:** abortion, United States, behavioral innovations, barriers to abortion access, Reddit, post-*Dobbs*

## Abstract

Following the leak of the Dobbs decision in 2022, abortion access in the United States has faced heightened barriers, including legal restrictions, financial constraints, and logistical challenges. In response, individuals seeking abortion care can employ innovative behavioral strategies to overcome these barriers and reshape their abortion experiences (ie, “behavioral innovations”). This paper explores the behavioral innovations to access abortion that people discussed and recommended within a geographically dispersed community of peers on an abortion-supportive Reddit community (r/abortion). Using a hybrid inductive and deductive thematic qualitative analysis approach with a purposive sample of comments in the r/abortion community in 2022 following the *Dobbs* leak (May-December, n = 131 comments), we identified discussion of abortion access innovations related to getting in-clinic care, self-managed abortion (SMA), funding assistance, privacy, and emotional support. Innovations included sharing online resources for clinic locations, specific travel recommendations to less restrictive states, accessing abortion medications through online services, and navigating the SMA process. Additionally, other less tangible innovations were discussed, including strategies for keeping abortions private and seeking emotional support. Our findings highlight how individuals within the r/abortion community discuss and share creative strategies for navigating the evolving barriers to abortion care. The r/abortion platform serves as a crucial resource for individuals seeking innovative solutions to these barriers, underscoring the need for diverse information-sharing practices to improve access to care as shifting legislation increasingly demands approaches beyond conventional norms.

**Highlights**

● Post-*Dobbs* decision, abortion seekers face growing legal, financial, and logistical barriers to abortion.

● Online communities like r/abortion on Reddit provide timely peer support and knowledge sharing amid increasing barriers to care.

● Commentors in r/abortion share diverse behavioral innovations – creative strategies for self-managed abortion, travel planning, funding navigation, privacy conerns, and emotional support.

● r/abortion serves as a vital platform for sharing experiences and guidance, helping others navigate barriers to abortion care and fill gaps left by traditional healthcare systems post-*Dobbs*.

## Introduction

Following the *Dobbs v. Jackson Women’s Health Organization (Dobbs)* Supreme Court decision in June of 2022, which revoked the constitutional right to abortion in the United States (US) and delegated the authority to regulate abortion to individual states, concerns about barriers to abortion access have grown significantly.^1^ Since *Dobbs*, nearly half of all US states have enacted bans or restrictions on abortion access as of October 2024.^2^ Faced with these challenges, people are adopting innovative approaches to access abortion services.^3-6^ These strategies combine traditional pathways with new methods, leveraging available resources to create effective pathways to accessing desired abortion care. Technologies and online tools play a key role, helping individuals discover, discuss, and implement these approaches.^7^ Platforms like Reddit serve as spaces for these discussions, where users share experiences, offer strategies, and engage with others’ posts and comments.^8^ This research aims to explore these discussions by analyzing comments shared on an abortion subreddit (r/abortion) following the leak of the *Dobbs* decision in 2022, providing insights into the various strategies people are employing to navigate the evolving landscape of abortion.

### Barriers to Abortion Access in the United States

Abortion is a common experience in the US, with 1 in 4 women having an abortion by age 45.^9^ While the Supreme Court decision *Roe v. Wade* protected the constitutional right to abortion in the US prior to 2022, access remained deeply unequal, shaped by state-level policies that created a patchwork of structural and social barriers, including abortion bans, gestational age restrictions, stringent facility requirements, and telehealth limitations.^10-12^ These challenges intensified in 2022, beginning with the unprecedented leak of Justice Samuel Alito’s draft opinion in *Dobbs v. Jackson Women’s Health Organization* in May. The final decision, issued nearly 2 months later, overturned *Roe v. Wade* and eliminated federal protections for abortion access.^13,14^

By 2024, nearly half of US states had enacted abortion bans or restrictions, many without exceptions for rape or incest, leading to widespread clinic closures and delayed care.^1,2^ Abortion bans prohibit access outright or allow it under narrowly defined circumstances, such as such as life endangerment, incest, or rape, while gestational age restrictions limit when in pregnancy a person can access care, often cutting off access before many people even know they are pregnant.^15^ Stringent facility requirements, such as requiring clinics to meet surgical center standards or physicians to have hospital admitting privileges, have forced the closure of many abortion providers.^16^ Telehealth limitations, particularly laws restricting medication abortion via telemedicine, further obstruct access, especially for those in rural or underserved areas.^14^

These restrictions increase travel distances, costs, and delays, often resulting in unattained abortions.^11,17-20^ Financial barriers, including procedural costs and expenses for travel, childcare, and lodging, further limit access.^21^ Restrictions also contribute to a rise in self-managed abortions, performed outside the formal healthcare system, limiting the autonomy of those seeking care.^10,15,17,22-24^ For those unable to access any form of abortion care, the consequences can be profound. Research has shown significant negative outcomes for individuals denied access to wanted abortion, including greater economic hardship, such as higher rates of unemployment and poverty. Denied individuals also face more health complications, such as infections and chronic pain, and report poorer overall health. Additionally, they are more likely to raise children alone and have children who face developmental challenges.^25^

The COVID-19 pandemic highlighted the potential for telehealth to improve access to abortion care. In April 2021, the US Food and Drug Administration (FDA) announced that it was lifting the in-person dispensing requirements for mifepristone, 1 of 2 medications used in a combined medication regimen abortion, the standard and most used regimen for medication abortion in the US.^26,27^ This has allowed patients in states where abortion is deemed an essential service to receive the medication by mail following a telehealth consultation.^28,29^ Today, despite demonstrated safety, efficacy, and feasibility, telehealth continues to face legal barriers, particularly in the provision of medication abortion. It is completely banned in 20 states, while 4 other states require an in-person visit as part of a hybrid telehealth model.^30,31^ Telehealth for medication abortion also faces broader legal threats, including challenges to both the FDA’s approval of mifepristone and the continued authorization of telehealth as a method for abortion care.^28^

Despite these restrictions, the number of abortions in the US increased in 2023 to over 1 million, the highest in more than a decade, with medication abortions accounting for 63% of all abortions, up from 53% in 2020.^28,32,33^ This increase is due in part to the growing availability of telehealth services for medication abortion.^27^ Legal battles continue to shape the accessibility of both telehealth and medication abortion, with ongoing threats and changes contributing to widespread uncertainty about the legality and availability of abortion care.

### Approaches to Navigating Barriers

Abortion seekers have historically adopted new strategies or behaviors to overcome access barriers,^7^ a concept we describe as “behavioral innovations.” Behavioral innovations are defined as one or a connected sequence of intangible problem-solving activities that provide the user or developer a functionally novel benefit relative to previous. Existing resources and tools, though not always novel, function as innovations when individuals discover and use them in a new way or for the first time. While not explicitly studied in the context of abortion in the US, behavioral innovations can address challenges such as restrictive laws, limited healthcare access, financial constraints, and social stigma.^34^ This study highlights behavioral innovations for abortion in the US, focusing on the r/abortion subreddit as a platform for sharing and developing behavioral innovations, facilitating access by sharing strategies and resources.

#### Accessing in Person Clinical Care

For many people, particularly low-income individuals and those in rural areas, accessing in-person abortion care may now require traveling across state lines due to barriers like abortion bans, clinic closures, waiting periods, and local provider restrictions.^20,27,35^ In the 100 days following the *Dobbs* decision, 66 clinics either closed or stopped providing abortion care, leaving 14 states without a single abortion provider.^36^ Since, the share of patients across the US traveling out of state for care has doubled, with 1 in 5 traveling in 2023 compared to 1 in 10 in 2020, and average travel distances have tripled.^19,27^

As these barriers persist and evolve, travel for abortion continues to serve as a crucial adaptation strategy. However, travel for abortion poses significant logistical, financial, and emotional challenges, including finding clinics, covering transportation and lodging costs, and arranging time off work or childcare.^17,37^ Post-*Dobbs*, patients face longer appointment wait times and increasingly narrow legal windows for obtaining care, particularly due to gestational age restrictions that prohibit abortion after a certain point in pregnancy.^38-40^ As of October 2024, 4 states, Florida, Georgia, Iowa, and South Carolina, have implemented laws that ban abortion at 6 weeks. These bans make timely access to care especially difficult, as over a third of people don’t realize they are pregnant until at or after 6 weeks. The need for travel often further increases the time it takes to access in-person care, making it even more difficult for patients to obtain services before they surpass these legal limits.^41^ Despite these obstacles, many continue to travel for essential care, underscoring not only the barriers created by restrictive laws, but the resilience of those seeking abortions and the importance of innovative approaches in navigating travel-related challenges.^27^

#### Abortion Funds

In 2022, procedural abortions were estimated to cost around $2,000, and in 2023, the average price for a medication abortion was $563.^11,42^ These figures do not account for additional costs associated with accessing abortion care, such as travel and childcare, which have risen significantly in recent years. According to The Brigid Alliance, an organization that arranges and funds travel and practical support for abortion seekers, the expenses for abortion access outside of the procedures, including childcare and travel, now exceed an average of over $2,000 since the *Dobbs* ruling in June 2022.^43^ Additionally, federal law prohibits Medicaid and other federal insurance programs from covering abortion care, except in cases of rape, incest, or when the pregnancy endangers the patient’s life. Only 17 states offer public funding for most abortion care.^44^ Consequently, nearly 70% of patients pay out-of-pocket. As 41% of abortion patients live below the federal poverty level (FPL) and 30% between 100% and 199% of the FPL, the lack of insurance coverage, coupled with significant costs of abortion care, creates significant financial barriers to care.^45^

Abortion funds help to address these financial barriers, providing assistance at national and regional levels for both direct and indirect costs. These funds, in place for decades, often cover an average of $1,000 per patient, with support ranging from $30 to $8,000. Many also help with travel, lodging, and logistical support, offering crucial aid for individuals navigating financial hurdles to care.^46,47^

#### Self-Managed Abortion

Self-managed abortions (SMA), also referred to as self-sourced or self-administered abortion, are defined for this research as when a person performs their own abortion outside of a medical setting, with or without clinical support.^48^ While SMA has been inconsistently defined across research, with increasing differentiation between SMA and telehealth or telemedicine abortion, the concept was defined broadly for this research to capture the plurality of abortion experiences outside of clinical contexts.^48-50^

Some people prefer to self-manage their abortion, seeking a SMA may be motivated by legal barriers, clinic closures, financial constraints, limited transportation, and stigma.^37^ SMAs can involve various methods, but medication abortion, using medications mifepristone and misoprostol that work to block pregnancy hormones and induce uterine contractions, is the only FDA-approved method and is considered safe and effective when followed by medical guidelines.^51,52^

SMAs have been used throughout history and across cultures.^24^ In the US, innovative ways to access SMA have arisen in response to abortion restrictions, particularly through telehealth and online platforms. Before the *Dobbs* decision, the lifetime prevalence of an SMA was estimated to be 7%, representing more than 1 in 4 of all abortions.^9,53^ This number has likely risen with the increase in legal barriers to clinical abortion access.^54^

As people seek SMAs, many turn to websites and services that facilitate access to abortion care and medications. Research shows that increased abortion restrictions correlate with a rise in internet searches for abortion medications.^23,55^ Various online platforms provide online access to medication abortion, such as Aid Access, a leader in the space, as well as HeyJane, Choix, and Abortion on Demand. These online platforms offer telehealth services and ship medications directly to users, making them vital for individuals facing legal, financial, or geographic barriers to abortion care.^48^

### Rationale and Research Questions

#### Reddit as a Tool for Abortion Seekers

In recent decades, Americans have increasingly taken proactive roles in managing their health, with the internet playing a central role in providing access to health information, services, and support.^8,56^ Online resources in the US play a significant role in providing widespread access to health information, services, and support. Online health communities, in particular, have the potential to improve health outcomes by offering a platform for people with common health interests to share experiences and information.^8^ Reddit, one of the most popular forum-based social networking sites, is used by roughly 1 in 5 adults in the US.^57^ As a forum-based platform, Reddit offers users the opportunity to engage in detailed, community-driven conversations, making it an ideal platform for people seeking or sharing information, advice, experiences, and support.^58^ Reddit also hosts many health-focused communities, including those addressing reproductive health topics like sexually transmitted infections, contraception, pregnancy, and abortion. Reddit’s anonymity makes it especially appealing for discussing stigmatized topics like abortion, enabling users to ask questions and share information they may not disclose elsewhere.^59,60^ Comments on these posts are also particularly important as they facilitate peer-to-peer support, provide diverse perspectives, and enhance the depth of information available through shared personal experiences and knowledge.^58^

The subreddit r/abortion, with over 56,000 members as of 2024, is a vital resource for individuals seeking or sharing abortion-related information. This pseudonymous community facilitates discussions on abortion decision-making, barriers to access, self-managed abortions, and personal experiences.^61,62^ Moderated for accuracy and safety, r/abortion offers geographically diverse and timely support, allowing members to connect and share advice.^63^ While previous research has examined various topics within r/abortion, little has focused on the role of comments.^61,62^ Comments are integral to innovation within this community, enabling real-time discussions that reveal emerging trends, offer personalized guidance, and present unique perspectives. Analyzing how comments are used provides deeper insight into the information exchange within r/abortion and how these discussions shape the experiences and strategies of those navigating abortion-related decisions, barriers, and access.

#### Research Questions

Given the changing context of abortion access in the US, increasing barriers, and the proliferation of technology used to access abortion-related information, services, and support, more people are using and discussing behavioral innovations to facilitate access to abortion. The purpose of this paper is to investigate and understand the dynamics of these innovations discussed in r/abortion, shedding light on how individuals creatively navigate restrictive environments to exercise their reproductive autonomy. Specifically, we sought to answer the following research questions based on comments shared in r/abortion following the *Dobbs* decision leak in 2022:

1. What new and/or different ways of accessing clinic-based abortion care did r/abortion community members share?
2. What funding and cost coverage innovations did r/abortion community members share?
3. What approaches to self-managed abortion (SMA) did r/abortion community members describe?
4. What other abortion access innovations did r/abortion community members share?

By examining the interactions and shared knowledge within r/abortion, we can gain valuable insights into how individuals are creatively overcoming obstacles to abortion access in the post-*Dobbs* landscape.

## Materials and Methods

### Data Collection and Pre-processing

The study utilized publicly available, near-real-time data from Reddit. The data collection approach was informed by prior studies that utilized PushShift’s Reddit Application Programming Interface (API) to analyze health-related discussions on the platform.^64,65^ From October 2022 to February 2023, our research team, all based in the US, gathered all posts from the r/abortion subreddit during this timeframe by integrating data from both PushShift’s Reddit API and the official Reddit API. After compiling the complete dataset, we excluded posts that were removed or deleted, contained only images or links, or had fewer than 30 characters. This refined dataset formed the basis for our analysis, with usernames and Reddit submission IDs removed to protect user anonymity.

After applying the basic exclusion criteria, we used Python’s random.sample function to randomly select 10% of posts from each month within the timeframe of qualitative analysis (5/02/2022-12/24/2022) to create a subsample of eligible posts (n = 523). To determine which of these posts were eligible for comment coding, we manually identified posts during the coding process that: (1) described experiences involving specific barriers to accessing abortion, (2) sought information or solutions to overcome barriers to abortion access, and (3) shared strategies or innovations for overcoming abortion access barriers. We also excluded posts from the qualitative sample if the author indicated they were living outside the US, whether by directly stating their location, referencing a non-US healthcare system (such as the National Health Service in England), or describing an abortion access experience that clearly reflected a different legal context than the US (such as abortion being completely banned nationwide). These posts were replaced with others from the same time period. The associated comment threads (n = 86) responding to the eligible posts were extracted for qualitative analysis. From the 86 comment threads, there were 131 comments describing innovations. These comments were organized based on their respective post threads, facilitating a contextualized thematic qualitative analysis for this purposive sample of comments on r/abortion following the *Dobbs* leak in 2022.

To address sample size adequacy, we drew on the concept of information power to guide our approach. Information power suggests that the more relevant and focused the information a sample holds in relation to the study aim, the fewer participants are needed.^66^ We believe that we had sufficient information in the sample of comments we analyzed to address the aims of this study, particularly given our clearly defined research questions, the specificity of the sample, and the use of a hybrid coding approach. Together, these factors indicate that our sample provided adequate information power for the purposes of this analysis.

### Analysis

Qualitative analysis was done using a hybrid deductive and inductive thematic approach to utilize previous research describing abortion experiences while allowing for the emergence of new and salient codes.^67^ The reliability of a priori codes was tested, the coding approach was refined, and the remainder of the submissions were coded. MAXQDA (VERBI software) was used to record coding done by the team, allowing 10% of coded submissions to be sent to an additional team member for open double-coding to facilitate reliability and consistency.^68,69^ The coding team collaboratively reviewed and resolved any coding inconsistencies, reaching a consensus on the most appropriate application of codes.

Following coding, additional analysis was done with all content coded as describing an abortion access “innovation.” This analysis defined innovation as “any approach to overcoming barriers to informed abortion decision-making and access.” This included child codes specific to self-managed abortion (SMA), the use of abortion funds or other sources of funding to support abortion access, information about new or different ways to access in-clinic abortion care, and any other behavioral innovations facilitating abortion access. For comments within each innovation code group, the content was reviewed, and a descriptive categorization process was used to catalog information, addressing the outlined research aims. Specifically, we cataloged (1) the innovation recommendation being made in the comment into categories defined for each type of innovation, (2) the specific resource(s) shared in the comment (eg, website link, abortion clinic, hotline number, etc.), (3) if the innovation recommendation was explicitly described as based on the personal experience of the commenter, and (4) if the commenter (including the original poster) indicated they planned to or had implemented the innovation. After categorization, notes were used to summarize findings, identify key themes, and summarize findings across themes.

**Figure 1.**
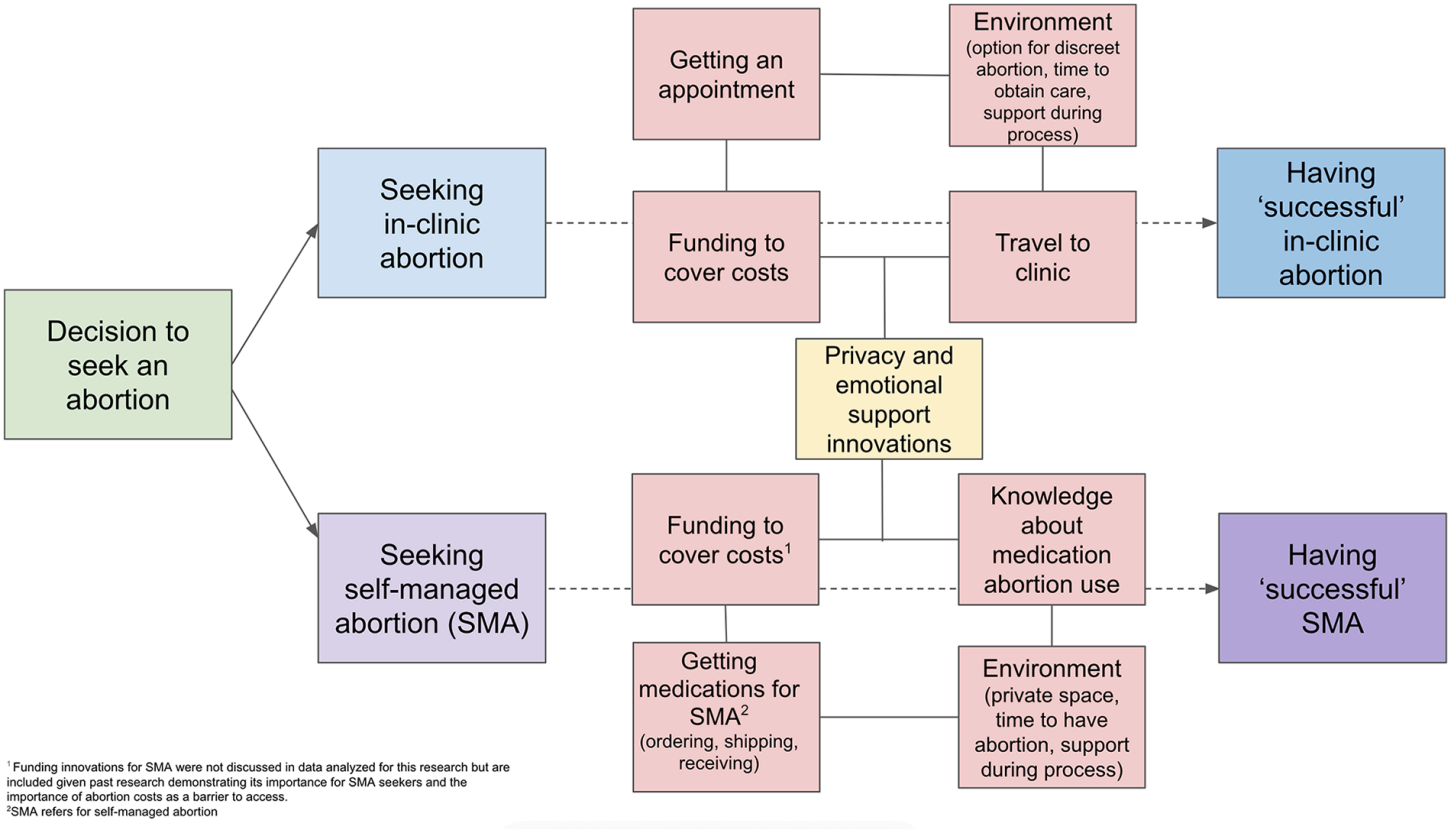
Conceptual framework outlining types of innovations shared in r/abortion comments by abortion modality.

During the summarization process, we found that the innovations shared were generally connected to the modality of abortion care being sought, generally providing resources to access either clinic-based abortions or SMA. As such, findings were grouped into innovations that sought to facilitate access to in-clinic abortion care, SMA, and “other” innovations not tied to a specific abortion care modality. A conceptual framework for the types of innovations shared to facilitate each type of abortion is presented in Figure 1, outlining how different innovations were shared to support different challenges or needs of r/abortion community members as they sought an abortion. Innovations helped r/abortion posters and commenters navigate various barriers to having a “successful” abortion, namely a complete, safe, and supported abortion.

### Ethical Considerations

This study was exempted from review by the Office for the Protection of Human Subjects at the University of California, Berkeley (2022-08-15585). Although the data were publicly accessible, ethical concerns regarding participant confidentiality and privacy should still be considered.^70-72^ These matters are particularly crucial due to the sensitive nature of abortion narratives.^73,74^ The current recommended approach when dealing with Reddit data involves implementing a “heavy disguise” process accompanied by rigorous testing to generate representative narratives while protecting the privacy of the community members.^75,76^ While all analyses were conducted using complete text from post and comment submissions, we employed an “ethical fabrication” method to create representative narratives that were vetted through Google, Reddit, and a plagiarism detection tool (duplichecker.com). All links in composite quotes are redacted and replaced with a summary description to reduce the identifiability of the original submitters. These composite quotes aim to represent the narratives shared in the r/abortion community without posing additional social or legal risks. Additionally, this study followed the Standards for Reporting Qualitative Research (SRQR) guidelines to ensure transparency and rigor in study design, analysis, and reporting.^77^

### Positionality Statements

In alignment with our epistemological framework, it is essential to critically reflect on and disclose our social locations as they relate to this analysis. This work has been conducted by an interdisciplinary collective of researchers and advocates, grounded in the principles of reproductive justice. Positionality statements for each team member are provided below to ensure transparency and contextualize our perspectives.

- [Author 1] is a female-identifying person who lived in an abortion-protective US state at the time of this research; she is a Reddit user who primarily reads content.
- [Author 2] is a female-identifying person who lived in an abortion-protective US state at the time of this research; she is a Reddit user who primarily reads content.
- [Author 3] is a female-identifying person who lived in an abortion-protective US state at the time of this research; she is a Reddit user who primarily reads content.
- [Author 4] is a female-identifying person who lived in an abortion-protective US state at the time of this research; she is a regular Reddit user.
- [Author 5] is a male-identifying person who lived in an abortion-protective US state at the time of this research; he is an active Reddit user.
- [Author 6] a female-identifying person who lived in an abortion-protective US state at the time of this research; she is not a Reddit user; she works on sexual and reproductive health in abortion-restrictive countries.
- [Author 7] is a female-identifying person who lived in an abortion-protective US state at the time of this research; she is a Reddit user who primarily reads content.

Our team had varying levels of experience using Reddit, which helped us engage with data from r/abortion in a thoughtful and informed way. Notably, conducting this research actually led some team members who had not used Reddit before to start exploring the platform in their own lives. While all researchers lived in abortion-protective areas at the time of the study, several of us are originally from or have worked in places with more restrictive abortion laws, and those personal experiences shaped how we analyzed the data, interpreted the results, and wrote up our findings.

## Results

The innovations described in the purposive sample of comments (n = 131), shared in response to posts sharing challenges, provided Reddit users with resources and novel approaches to navigating the changing abortion access landscape. Specifically, comments described innovations facilitating access to in-clinic abortion care (n = 52 comments), funding and cost-coverage innovations (n = 12), innovations related to SMA (n = 45), and privacy or emotion-related innovations (n = 28). These innovations are presented in the following sections in alignment with the outlined specific aims.

### Accessing in-clinic Abortion Care

In comments of r/abortion, users share innovations to assist in accessing clinic-based abortion care in 52 comments. These comments most commonly included information on online resources, clinics, and travel recommendations. The most prominent online resources include abortionfinder.org, ineedana.com, and providecare.org. Generally, these online resources were shared to help r/abortion users find clinic information and search for abortion providers, often in less restrictive states than where they lived. People shared this information in comments:

*“You should call clinics to see if they have any earlier appointments. You can look at [popular abortion finder website 1] and [popular abortion finder website 2] to see a list of clinics that might work for you.”* (composite quote)

Innovations also describe specific clinic recommendations for those seeking in-person abortion care. The clinics suggested included specific Planned Parenthood locations and various independent abortion clinics, generally shared based on the self-described location of the person seeking care. Comments discussing clinics described clinics as “good,” “supportive,” and “non-judgmental,” sharing positive impressions or personal experiences:

*“If you can travel, I really suggest you go to [specific clinic name]. The staff is really kind and understanding and genuinely pro-choice.”* (composite quote)

In addition to sharing existing clinics and online resources, comments also shared specific travel recommendations to access in-clinic care. While some comments involved a general suggestion to travel to another state, many shared suggestions of specific states or cities to travel to, with New Mexico, Kansas, Colorado, and Illinois being most commonly suggested. Comments generally shared destinations based on abortion legality and proximity to abortion-restricted states in which commenters indicated they resided, as well as positive experiences with specific clinics in these areas. These abortion destinations were often described as “safe,” “easy,” “close,” with commenters also sharing accessibility and care options in these locations:

*“I’m also in Texas and think that New Mexico is the safest state for accessing care. You’re able to get seen the same day, and even if it is expensive to travel, it may be the best option if you know it’s what’s right for you.”* (composite quote)

Notably, one comment from a post’s original poster shared their intent to travel to a clinic in another state based on the recommendation of another commenter:

*“I’m going to take this advice and travel to a clinic in a different state. Thank you so much for your help!”* (composite quote)

As comments frequently offered guidance uniquely suited to the poster’s stated region, whether by recommending resources to locate nearby clinics or suggesting accessible states with fewer restrictions, this location-specific advice allows users to find resources that are both relevant and potentially practical based on their geographic and legal circumstances.

### Funding for Accessing in-person Care

Among comments recommending innovations to access in-person care, 12 comments also referenced funding related to abortion access, including the costs associated with travel for abortion care and other abortion-related costs. These comments shared resources for funding interstate travel costs as well as options for covering abortion procedure costs. These innovations were generally in the form of abortion funds, clinics, and online funding resources, with specific regional and state abortion funds being the most commonly recommended. When recommending abortion funds, commenters suggested reaching out to these organizations directly and inquiring about aid options:

*“You should call [independent abortion clinic name] to ask for help. They might be able to provide you with funding with their [clinic-based abortion fund]. If not, you should also look into the [state abortion fund and regional abortion fund names], who might be able to get you funding for any travel you might have to do.”* (composite quote)

These commenters discussed the critical role that financial resources play in facilitating access to clinic-based abortion services, particularly in the context of navigating various legal and logistical barriers to access care in-person. Some commenters emphasized the intertwined necessity of securing funding for clinic-based abortion care, while also noting that access to care is essential to obtain that funding:

*“There is a clinic in Jackson, TN open, but I don’t think they have any more available appointments. If you’re able to travel to other states like Georgia or Florida, you might be able to find some funding resources for these states on [link to website providing location-specific abortion resources]. But you can only get funding if you make a clinic appointment, so do that first.”* (composite quote)

Sharing online resources to find funding support was also common. The most prominent online resources shared include ineedana.com and abortionfinder.org. These resources were recommended as avenues for users to be connected to funding information, often allowing them to search for funding based on their location and relevant to their specific circumstances. Comments that recommended funding resources did not mention whether these resources had been used by the commenters or the original poster.

### Self-Managed Abortion (Explicit and Inferred)

To assist those navigating barriers to abortion access, commenters on the r/abortion page often suggested innovations related to SMAs, with 45 comments describing the use of SMA. Discussions of SMA ranged in topic, including comments about ordering from a specific platform, the shipping process, receiving a package containing pills for a medication abortion, and the process of using abortion medications without clinical supervision. Innovations that commenters suggested generally sought to either help others acquire or access medication abortion pills for SMA or to navigate the process of self-managing an abortion.

Among comments discussing SMA, 32 specifically discussed medication abortions, with most of those comments referring to online resources for the direct purchase of abortion medications for self-management. Aid Access, a nonprofit that provides access to abortion medication by mail, was the most commonly suggested online resource. Other resources included OnlineAbortionPillsRX, HeyJane, Abortion on Demand, and Juniper Midwifery. Comments discussing these resources often introduced these specific sites while also confirming of their validity–either generally or based on personal experience using the platform:

*“From recent experience, they’re legit. I don’t blame you for being skeptical, I was too! If you google AccessAid, they have their own Wikipedia page and lots of articles. Some of the articles aren’t friendly, but none of them mention fraud/lying on their part. That’s how I made the choice to trust them, and I’m glad I did. It’s such a relief to have it completely confidential in my own home.”* (composite quote)

In addition to innovations for directly accessing abortion medications, 14 comments provided resources and innovations for navigating the actual process of a SMA, both before and after pills were acquired. These comments included information about the ordering process and what to expect, shipping time and reassurance in the process of waiting for pills to arrive, the need for discreet packaging, and specific instructions for taking pills and what to expect during the process of having a medication abortion. PlanC.org, an organization and online platform providing information on self-managed abortions, was recommended in 12 comments, where commenters suggested their site as a source for additional information.

Additionally, comments included actionable innovations to help navigate certain challenges during self-managed abortions, such as medication administration and nausea management. Comments sharing these innovations included suggestions such as using a tampon applicator to insert abortion medication vaginally and specific over-the-counter medication to take during the process for nausea and pain. Since these comments focused on self-managed abortion (SMA) and thus addressed abortion outside traditional medical settings, the exchanges of information provided r/abortion users with access to recommendations, resources, and insights that might not typically be accessible through conventional healthcare channels.

Comments about SMA innovations were often contextualized by commenters’ own experiences, sharing their stories as a means to spread information and build credibility for their recommendations. Many commenters mentioned personal implementation of their recommended innovations. These comments not only served to confirm the effectiveness of commenters’ advice but also validate the experiences of others seeking SMA.

### Privacy and Emotional Innovations

Innovations discussed in comments also involved the discussion of other behavioral innovations that assisted r/abortion users in navigating barriers to abortion access. These 28 additional comments addressed serious concerns for those accessing abortion, including the need to keep their abortion private and seeking emotional support.

Comments discussing innovations that specifically addressed abortion privacy were common. These innovations specifically responded to users’ need to keep their abortion procedures or abortion travel a secret from either those around them or from legal authorities. Further, a major theme among these privacy innovations was either disguising the entire pregnancy and/or the abortion, or specific elements of the abortion process. The most commonly suggested privacy innovation was disguising an abortion as a miscarriage. These comments specifically suggest that those wishing to keep their abortion a secret could do so by telling others they instead miscarried because both peers and medical professionals will not be able to tell the difference.

*“A procedural abortion is easier to keep a secret because it will be fully done by the time you leave your appointment. You can also hide it if you tell your family you were just at another doctor’s appointment and discovered you had a miscarriage.”*(composite quote)

Other examples of privacy innovations include disguising travel for abortion care as a vacation. In addition, comments made suggestions for protecting users’ privacy related to technology use and digital tracking. These innovations include turning off tracking applications and enabling privacy settings on personal devices and leaving phones at home entirely when traveling for abortion.

Other behavioral innovations included emotional support innovations making suggestions for users to find support in navigating the unique emotional challenges when accessing abortion and navigating barriers. Comments suggested accessing specific support services, including therapists, hotlines, online resources, and reading materials. These comments shared information similar to the following:

*“Many resources, including this subreddit, therapy, supportive family and friends, helplines like [specific abortion helpline], and various other reproductive rights communities are all here for you and cheering you on in your path to healing. If you’re interested in more support, I recommend checking out [specific abortion helpline’s] resources page, where they suggest a book.”* (composite quote)

While most of the privacy and emotional innovations suggested were untested by commenters based on their submission, some commenters mentioned the implementation of these behavioral innovations in their own lives, with stories such as:

*“When I was 6 weeks pregnant, I experienced some spotting. While the spotting eventually stopped, the doctor reassured me that it was ok and I was still pregnant. Nevertheless, I informed my family that the bleeding had worsened, and I sadly lost the baby. In truth, I chose to terminate the pregnancy, but they will never know the truth unless I choose to share it with them.”* (composite quote)

While these innovations were less tangible compared to the other behavioral strategies discussed, they were a common response among users of r/abortion to significant concerns about maintaining privacy during abortion process and the need for emotional assistance.

## Discussion

Our findings highlight the vital role of online communities like r/abortion in providing timely information and support for those navigating abortion access. In the absence of comprehensive education and services, these platforms fill critical informational gaps. While past research has examined posts about access challenges,^61^ our study of subreddit comments reveals narratives of innovative strategies shared and used to overcome barriers. These platforms and their contributors offer tailored, timely assistance that addresses the complex needs of abortion seekers, especially amid shifting legal and social landscapes. Understanding how they foster innovation is essential for shaping responsive healthcare policies and services.

Our study highlights the crucial role Aid Access played in self-managed abortions among r/abortion commenters following the 2022 *Dobbs* leak. Nearly every comment discussing self-management mentioned Aid Access, underscoring its importance alongside other online resources. Since 2018, Aid Access has been the first physician-led service to support SMA by providing abortion medications by mail in all 50 states, offering medications, and instructions on a sliding scale or for free.^39^ In its first 2 years, Aid Access received 57 506 requests, providing medication abortion to thousands despite operating outside the formal healthcare setting.^78^ Our findings align with past research on the rise of SMA requests post-*Dobbs*, driven by factors such as legal confusion and clinic disruptions.^54,78,79^ This trend highlights the growing reliance on services like Aid Access due to limited formal abortion access.

While Aid Access was the most discussed resource, other platforms like HeyJane, OnlineAbortionPillsRX, Abortion on Demand, and Juniper Midwifery were also recommended. Research on these platforms is limited, but their numbers have grown significantly since the *Dobbs* decision.^54^ These platforms vary in services, geographical reach, cost, reliability, and user experience, offering diverse options for SMA.^80^ Examining users’ experiences with these resources could reveal best practices and areas for improvement, informing more responsive and equitable reproductive healthcare approaches.

Our findings also shed light on the dynamics of travel for abortion care as described in r/abortion post *Dobbs*. Previous research has shown that since the *Dobbs* ruling, the number of patients traveling out of state for abortion care has doubled, and average travel distances have tripled.^19,27^ Our research findings qualitatively align with these trends, as many comments described the need to not only travel long distances to access care, but also plan intentional interstate travel potentially spanning hundreds of miles, often from restrictive states to those where abortion was more accessible. Commenters recommended specific states and clinics as ideal destinations, with certain states emerging as the most recommended destinations for abortion services based on people’s locations and needs.

Discussions around travel options often intertwined with conversations about funding support, with abortion funds emerging as a key resource. Commenters shared instructions, recommendations, and specific funds based on users’ locations, offering tools to help people navigate the complex abortion fund landscape across the US. While our study does not include demographic information, previous studies have shown that those who use abortion funds are primarily young, single, African American, low-income, and seeking funding for second-trimester abortions.^46,81^ Individuals seeking funding often face significant economic challenges, such as unemployment, reliance on public assistance, and homelessness, making abortion fund access vital for overcoming these barriers.^46^ As abortion fund assistance is especially critical for marginalized groups, r/abortion comments sharing this information highlights the role of online communities in providing personalized, timely strategies to support those most in need. Future research should examine the evolving role of abortion funds in supporting individuals navigating logistical and financial barriers to abortion access, including travel decisions, as the post-*Dobbs* landscape shifts. Abortion funds are often location-based and not universally accessible, highlighting the need to map their availability. Understanding how people navigate interstate travel and access funding is essential for informing policies and interventions to improve abortion access and address gaps in support systems.

Concurrent with the need for abortion seekers to access care, r/abortion commenters also discuss the need to employ innovative strategies to maintain privacy in their access to this care. While these types of innovations were less commonly suggested and less easily categorized than the innovations to access clinic-based care or SMA, these “other innovations” served as equally vital resources for people in r/abortion navigating abortion barriers.

Previous research has examined the role of privacy in abortion decision-making, particularly in contexts where legal restrictions and social norms shape disclosure choices. Many individuals seeking abortion care express concerns about maintaining anonymity, whether due to legal ramifications, fears of judgment, or the desire to control personal information.^82^ Studies indicate that two-thirds of women anticipate negative consequences if their abortion becomes known, with 58% feeling the need to keep it secret from friends and family.^83^ The discussions in r/abortion highlight how pseudonymous platforms serve as a critical space for sharing advice and information while mitigating privacy risks, an increasingly pressing concern as legal landscapes continue to shift. Past research has shown that digital platforms that allow users to remain anonymous offer a safer environment for individuals to access abortion-related support, protecting them from both legal and social risks.^84^

Further, our analysis revealed a pronounced need for emotional support among individuals navigating abortion-related privacy concerns based on comments shared in the r/abortion community. Similar to the need for privacy, the need for emotional support is not met for many abortion seekers due to the current abortion landscape, characterized by stigma surrounding abortion and legal restrictions that limit access to resources, discourage open conversations, and create fear of repercussions for those seeking help. Thus, platforms like r/abortion emerge as unique resources offering emotional support in a pseudonymous community of peers, addressing the emotional needs of abortion seekers.

Unlike more tangible resources, such as online abortion funds or informational websites, these privacy and emotion-focused behavioral innovations are less accessible through traditional information-seeking methods like search engines or online guides. However, r/abortion allows users to share these types of intangible resources, often based on their own experiences implementing the innovation. This again speaks to the unique strengths of the r/abortion community as a responsive resource, providing unique tools to preserve privacy, wellbeing, and autonomy in the face of legal risks.

Past research shows that online communities provide crucial social support in healthcare by offering spaces for individuals to share experiences, seek guidance, and access peer support.^85^ However, little attention has been given to their role in addressing abortion-related challenges and fostering innovation. Our findings highlight the significant role these online spaces play in providing abortion seekers with information and support beyond what traditional healthcare offers, facilitating access to resources needed to navigate barriers. Reddit, in particular, has become a vital tool for disseminating information and support across geographically dispersed communities of peers, allowing users to share and seek experiences, discuss options, and provide practical guidance on navigating legal, financial, and logistical challenges related to abortion access. By facilitating the sharing of experiences, resources, and emotional support, online communities act as vital complements to traditional healthcare services, bridging accessibility gaps and addressing the complex challenges faced by abortion seekers.

Future research should further explore how online communities and social networking platforms can be used effectively as information provision tools in abortion and reproductive healthcare more broadly. Key areas of focus could include assessing the accuracy and reliability of information shared on these platforms, understanding how online communities affect decision-making and perceptions about abortion, and evaluating the impacts of online support networks on access to in-person reproductive care. Addressing these areas allows for a deeper understanding of the broader implications of technology, online communities, and social networking platforms in reproductive health, particularly in environments where traditional access to care is restricted. This understanding will be crucial for guiding future policies and health interventions, especially as abortion restrictions continue to evolve and more people turn to digital platforms for support.

### Strengths and Limitations

This research offers valuable insights into the experiences of individuals following various paths to access abortion, including people who did not seek care at an abortion clinic. By exploring discussions of innovative behaviors to facilitate abortion access in comments on an abortion subreddit following the *Dobbs* leak, our findings broaden our understanding of abortion experiences across different contexts during a time of extreme challenge and uncertainty for abortion access in the US. However, this research has limitations that should be considered. First, while the pseudonymity of Reddit and r/abortion created an appealing environment for people to discuss sensitive experiences that they might not share elsewhere, this anonymity limits our research process and conclusions. A significant limitation is the lack of demographic information, which prevents analysis of how experiences may vary by age, socioeconomic status, or other factors. Younger individuals and people of color, for example, are more likely to seek abortions later, face financial hurdles, and utilize abortion funds, but these nuances cannot be fully explored in this study.^46^ Further, the anonymity of the participants on r/abortion gives rise to ethical concerns surrounding the difficulty of obtaining informed consent. This anonymity means that personal experiences are utilized without formal attribution or explicit permission. Composite quotes were used to address this concern, rewording comments to maintain data integrity and protect user anonymity. However, in using this ethical fabrication process, there is a risk of altering the original meaning or context of the shared experiences, potentially impacting the accuracy of the research findings.

All data was user-generated and voluntarily shared based on individual interests, providing unique, inductive insights into users’ experiences, needs, and recommendations regarding abortion access in the US during the study period. However, users may have omitted details or presented experiences differently than in more objective research methods. While we excluded posts that clearly reflected non-US contexts, some posts where the author’s location was ambiguous may have been included. As a result, a small number of non-US-based experiences may be represented in the sample. To address this, we flagged potentially ambiguous cases during the coding process and discussed them as a team to determine whether the context aligned with the US legal and healthcare environment post-*Dobbs*. This collaborative review aimed to ensure the inclusion of only relevant, contextually appropriate posts. Additionally, the inability to track users’ actions post-comment limits understanding of the long-term impacts of shared innovations, as commenters may not report whether they followed through with recommendations, such as ordering abortion medications. While the study cannot systematically determine these impacts, comments suggesting implementation highlight the potential influence of the r/abortion community. Despite these limitations, this research offers valuable insights into the experiences of individuals seeking abortions post-*Dobbs*, especially those not using traditional clinic-based services.

## Conclusion

Our findings highlight the critical role of online communities like r/abortion in navigating barriers to abortion access, especially in the shifting landscape of reproductive healthcare following the *Dobbs* leak. These platforms facilitate the sharing of innovative strategies, from tangible resources like trusted abortion clinics, self-managed abortion options, and funding assistance to behavioral approaches like maintaining privacy and seeking emotional support, often overlooked in traditional healthcare settings. By sharing experiences and recommendations, users help others navigate the complexities of accessing care, filling gaps left by formal systems. These innovations empower individuals to manage their care while exposing the limitations of existing support structures. Understanding how these strategies are shared within online communities is essential for adapting abortion care to the evolving needs of seekers and improving access amid growing legal and social challenges.
